# Hyper-Chaotic Color Image Encryption Based on Transformed Zigzag Diffusion and RNA Operation

**DOI:** 10.3390/e23030361

**Published:** 2021-03-17

**Authors:** Duzhong Zhang, Lexing Chen, Taiyong Li

**Affiliations:** School of Economic Information Engineering, Southwestern University of Finance and Economics, Chengdu 611130, China; zhangduzhong@swufe.edu.cn (D.Z.); clx220081203001@smail.swufe.edu.cn (L.C.)

**Keywords:** hyper-chaotic, ribonucleic acid, color image encryption, transformed Zigzag

## Abstract

With increasing utilization of digital multimedia and the Internet, protection on this digital information from cracks has become a hot topic in the communication field. As a path for protecting digital visual information, image encryption plays a crucial role in modern society. In this paper, a novel six-dimensional (6D) hyper-chaotic encryption scheme with three-dimensional (3D) transformed Zigzag diffusion and RNA operation (HCZRNA) is proposed for color images. For this HCZRNA scheme, four phases are included. First, three pseudo-random matrices are generated from the 6D hyper-chaotic system. Second, plaintext color image would be permuted by using the first pseudo-random matrix to convert to an initial cipher image. Third, the initial cipher image is placed on cube for 3D transformed Zigzag diffusion using the second pseudo-random matrix. Finally, the diffused image is converted to RNA codons array and updated through RNA codons tables, which are generated by codons and the third pseudo-random matrix. After four phases, a cipher image is obtained, and the experimental results show that HCZRNA has high resistance against well-known attacks and it is superior to other schemes.

## 1. Introduction

Nowadays, rapid developments of Internet and digital technologies have led to tremendous digital multimedia contents transmitting over Internet networks. Thus, protection on the contents of digital data has attracted serious concern from medical, military, and many other areas. Various image encryption methods have emerged by using cryptographic techniques [1,2,3,4]. Although there exists a view that AES is not suitable for image encryption, Zhang recently refuted it by using AES of cipher block chaining mode to encrypt images [5].

The chaos-based encryption method has become one of the most ideal methods, since it has a lot of appropriate characteristics, e.g. high sensitivity on initial conditions, mixing property, ergodicity, complex behavior, etc. [6,7,8]. As a result, a lot of researchers have presented plenty of image encryption schemes with a chaotic system [9,10,11,12,13]. In [14], Askar et al. proposed a chaotic economic map based image encryption method, whose simulation results indicated that the proposed algorithm could successfully encrypt and decrypt the images, and it had a good performance on security tests, except noise attacks analysis. By using a single round based hyper-chaotic system, Shaikh et al. presented a color image encryption method with bi-directional pixel diffusion [15]. Additionally, Li et al. presented a "transforming-scrambling-diffusion” model based color image encryption method with a four-dimensional (4D) hyper-chaotic system, which could convert pixel values to gray format before scrambling [16]. There is no doubt that some of the encryption methods in these chaos-based schemes still have weaknesses to some extent. However, different chaotic systems are neither superior nor inferior each other. A high-dimensional chaotic system has complex chaotic behaviors with high time cost, while a low-dimensional chaotic system is opposite [17,18,19]. Hence, in this paper, a 6D hyper-chaotic system is employed as a pseudo-random numbers sequence generator for more complexity.

Zigzag is a common scrambling operation in image encryption [20,21]. In [22], Li et al. presented a 3D logistic map based color image encryption method with Zigzag scramble; the experiments showed that this method had brute-force attack and statistical attack resistance, but differential attacks analysis was missing. While, Wang et al. proposed a color encryption method with a Zigzag transformation, which could change the start pixel from upper left corner to the other three corners in an image [20]. Next year, Wang et al. [23] presented another image encryption method, which introduced an extended Zigzag confusion for a non-square image. Additionally, in [24], Zhao et al. proposed a novel color image encryption by combining Zigzag map and Hénon map together for permutation. However, these image encryption schemes implement Zigzag scramble on 2D images, which leads to some adjacent values in special positions of the image not being able to be scrambled, and different channels of a color image could not be scrambled, either. On the other hand, some image encryptions transformed 2D image to 3D cube [25], which gives out a new encryption inspiration on permutation, but most of them were focused on rotation, but not Zigzag. Therefore, Zigzag is utilized in diffusion on a 3D cube instead of scramble on 2D image to eliminate these drawbacks in this paper.

Deoxyribonucleic acid (DNA), a biological concept, has recently become a popular trend in the image encryption field [26,27]. By using DNA-based techniques, cipher images could obtain competitive entropy, correlation coefficients etc. [4,28,29,30,31]. In [29], Chai et al. presented a new diffusion mechanism that is based on the random numbers that are generated by plaintext image, and incorporated DNA encryption with four-wing hyper-chaotic system. Reference [32] proposed an image encryption method using a spatial map based DNA sequence matrix. In general, the DNA-based encryption mechanism includes two steps: use DNA operation rules to convert pixels of plaintext image to DNA codon matrix and change chaotic sequence to DNA keys to generate cipher image with DNA codon matrix.

While unlike the two strands structure of DNA sequences, Ribonucleic acid (RNA) is a single strand structure. RNA could form double helixes with complementary base pairing. By using this feature, some new image encryption methods have been proposed. In [33], Mahmud et al. presented an image encryption method by combining RNA with Genetic Algorithm (GA) through using a logistic map. In [34], Abbasi et al. employed Chen’s chaotic system to encrypt an image with imperialist competition algorithm and RNA operations. Yadollahi et al. utilized the concepts of DNA and RNA to construct a two-phase image encryption method [35]. While an image encryption method is presented by Wang et al. through using an one-dimensional (1D) chaotic system combined from Logistic and Sine map, extended Zigzag confusion, and RNA operation [23]. However, all of these four schemes focus on gray image encryption. Although there is a color image experiment in [23], it is realized by running the scheme three times in three channels.

Being motivated by above discussions, a novel color image encryption method, called HCZRNA, is proposed in this paper. At the beginning, a 6D hyper-chaotic system is employed to generate three pseudo-random matrices. Subsequently, one of the pseudo-random matrices is used to permute plaintext color image. Additionally, 3D transformed Zigzag diffusion is implemented on initial cipher image with the second pseudo-random matrix. After diffusion, an RNA operation is used to convert the diffused image to RNA codons array, and update this array through RNA codons tables that are generated by the third pseudo-random matrix. Finally, a cipher image is obtained.

The main contributions of this work is listed as follows:

- A novel 6D hyper-chaotic system is employed in this paper to produce chaotic matrix for permutation, diffusion, and RNA operation.
- A new 3D transformed Zigzag diffusion scheme is proposed to encrypt color images.
- RNA operation is modified specifically for color images.
- Extensive experiments and analyses demonstrate that the proposed HCZRNA could resist various types of attacks.

The rest of this paper is structured as follows: Section 2 introduces the used 6D hyper-chaotic system, 3D Zigzag and RNA. Section 3 presents the HCZRNA scheme and explains how initial values and pseudo-random matrix are generated in detail. Section 4 reports and analyzes the experimental results. Finally, Section 5 concludes this paper.

## 2. Preliminaries

### 2.1. The 6D Hyper-Chaotic System

There are a lot of classical chaotic systems, e.g. Sine map, Logistic map, Tent map, etc., which have simple mathematical forms and can be implemented easily. However, they suffer from small key spaces, predictable orbits, limited ranges, etc. Existing research has shown that higher dimensional chaotic systems are much securer for image encryption [36]. Therefore, a novel 6D hyper-chaotic system is employed in this paper for chaotic sequences generation, which could be described as Equation (1) [37].

(1) $$\begin{array}{cc} {\dot{x}}_{1} & =g\left(\omega +\beta x_{6}^{2}\right)x_{2}-ax_{1}\\ {\dot{x}}_{2} & =cx_{1}+dx_{2}-x_{1}x_{3}+x_{5}\\ {\dot{x}}_{3} & =-bx_{3}+x_{1}^{2}\\ {\dot{x}}_{4} & =ex_{2}+fx_{4}\\ {\dot{x}}_{5} & =-rx_{1}\\ {\dot{x}}_{6} & =x_{2} \end{array}$$

where a,b,c,d,e,f,g,r,ω, and β are controlling parameters, and $x_{i}\left(i=1,2,\cdots ,6\right)$ are state variables.

**Figure 1:**
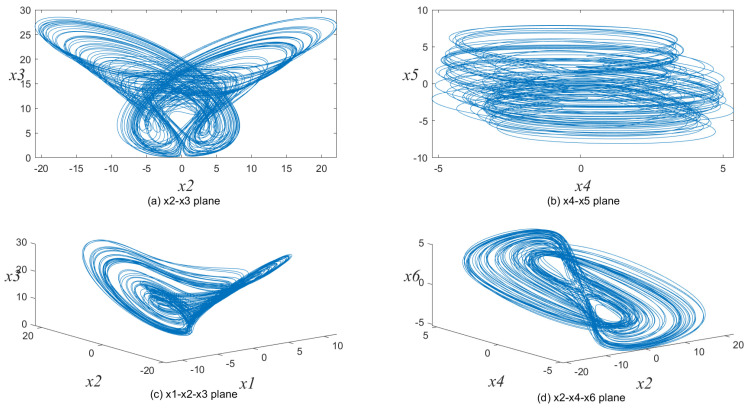
The attractors of six-dimensional (6D) hyper-chaotic system.

The fourth-order Runge–Kutta method is used to solve this hyper-chaotic system with step size h=0.001. We set the controlling parameters as (a,b,c,d,e,f,g,r,ω,β)=(0.3,1.5,8.5,−2,1,−0.1,0.9,1,1,0.2) and initial state variables as $\left(x_{1},x_{2},x_{3},x_{4},x_{5},x_{6}\right)=\left(0.1,0.6,0.2,0.02,1,0.5\right)$; Figure 1 shows this 6D hyper-chaotic system’s attractors. Its Lyapunov exponents are $\lambda_{1}=7.340$, $\lambda_{2}=0.087$, $\lambda_{3}=0.006$, $\lambda_{4}=-0.368$, $\lambda_{5}=-1.349$, $\lambda_{6}=-67.426$. Since this chaotic system has three positive Lyapunov exponents, its prediction time should be longer than other chaotic systems and it is hard to crack. Besides, this hyper-chaotic system exhibits limit cycles, quasiperiodic, and bursting behavior. Accordingly, it could generate effective a pseudo random sequence. More detailed demonstration could be found in reference [37].

### 2.2. 3D Transformed Zigzag Diffusion

Zigzag is a scanning method that is used to scramble pixels in image encryption. By scanning and taking a pixel with left upper corner of image, then taking other pixels one-by-one through Zigzag path, the image could be converted to a matrix in a fixed way. Hence, the image’s pixels could be scrambled.

**Figure 2:**
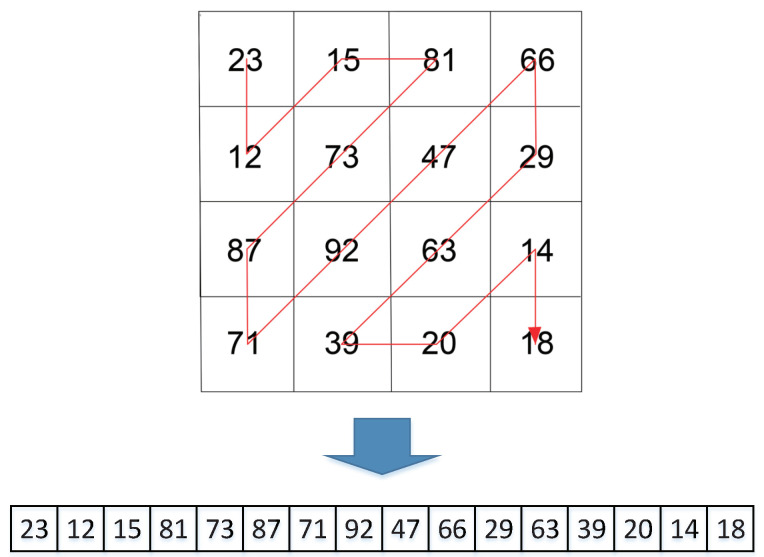
4×4 traditional Zigzag scramble.

**Figure 3:**
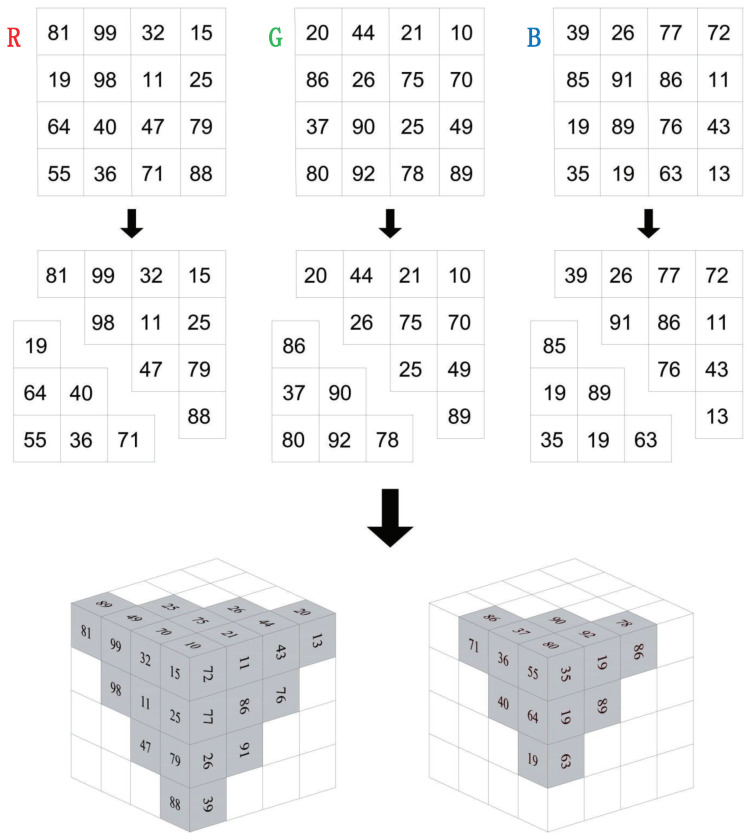
Color image to cube.The first row is three channels of a color image. The second row is the triangles generated from image. Additionally, the third row is the placement of triangles on a cube.

**Figure 4:**
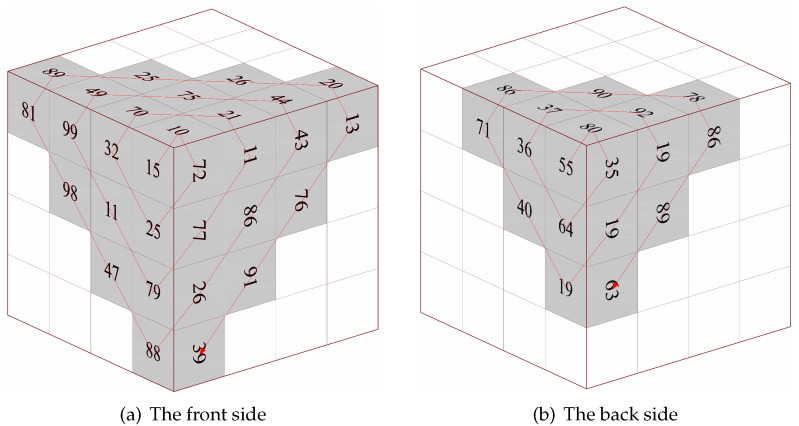
Three-dimensional (3D) transformed Zigzag diffusion. (**a**) is the Zigzag diffusion process on the front side of cube. (**b**) is the Zigzag diffusion process on the back side of cube.

**Figure 5:**
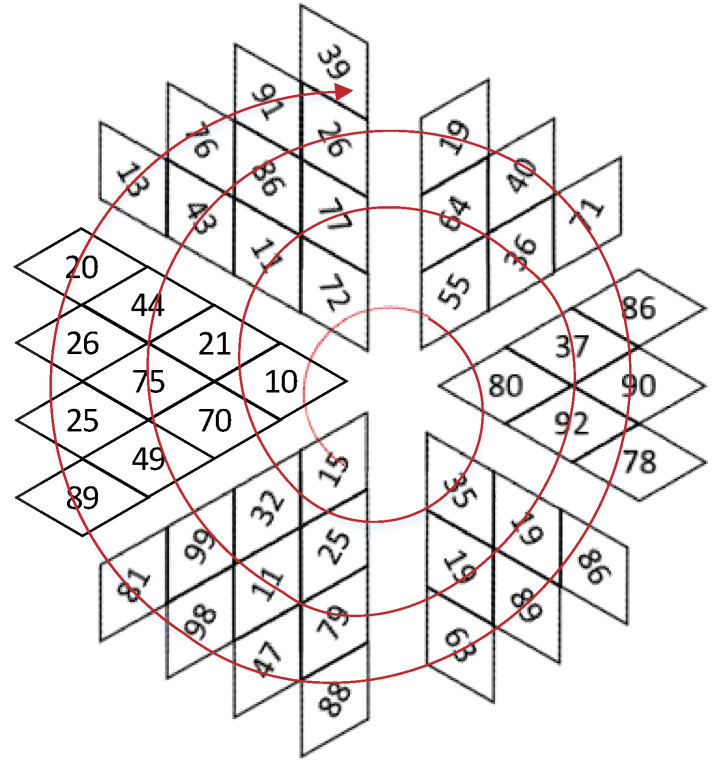
3D transformed Zigzag path. For all triangles on the cube, 3D transformed Zigzag diffusion is implemented through this order.

Traditional Zigzag scrambling could only walk through numbers in N×N matrix with a fixed Zigzag path; an example of 4×4 matrix is shown in Figure 2. By this fixed path, different channels of color image could not be scrambled with each other. Due to its drawbacks, this paper proposes a novel 3D Zigzag transformation. Using this transformation, each channel of color image would be cut into two triangles through a diagonal line, and be placed on opposite surfaces of a cube, which is illustrated by an example of 4×4×3 matrix, as shown in Figure 3. Subsequently, diffusion would start from origin vertex of the frontal side of the cube, and walk through every pixels on six surfaces at the front and back side synchronously with spiral Zigzag path, as in Figure 4. For all triangles that are placed on the cube, the order of diffusion is shown in Figure 5. In this way, different channels of color image could be diffused together.

### 2.3. RNA Operation

RNA is one of the major macromolecules necessary for living organism. RNA has a single strand structure with four nitrogen bases: adnine (A), cytosine (C), guanine (G), and uracil (U). For these four units of RNA, a binary system could be employed for representation, which is shown in Table 1. According to the base pairing rules, four bases of RNA could be coded and constructed into three nucleotides that correspond to one amino acid called codon. Accordingly, there are 64 codons truth table of bases combinations, as shown in Table 2. Assuming that pixels in the image could transfer into six-bits format, a corresponding RNA codon could be found in Table 2.

## 3. Encryption and Decryption

In this paper, image encryption could be divided into three parts. Firstly, a 6D hyper-chaotic system is employed to generate chaotic matrices for encryption processes. Subsequently, three-dimensional (3D) transformed Zigzag diffusion is implemented on the permuted image. Finally, RNA concept is used for encoding and decoding.

### 3.1. Encryption Scheme

Suppose that plaintext image has *N* rows and *N* columns with RGB channels.

**Figure 6:**
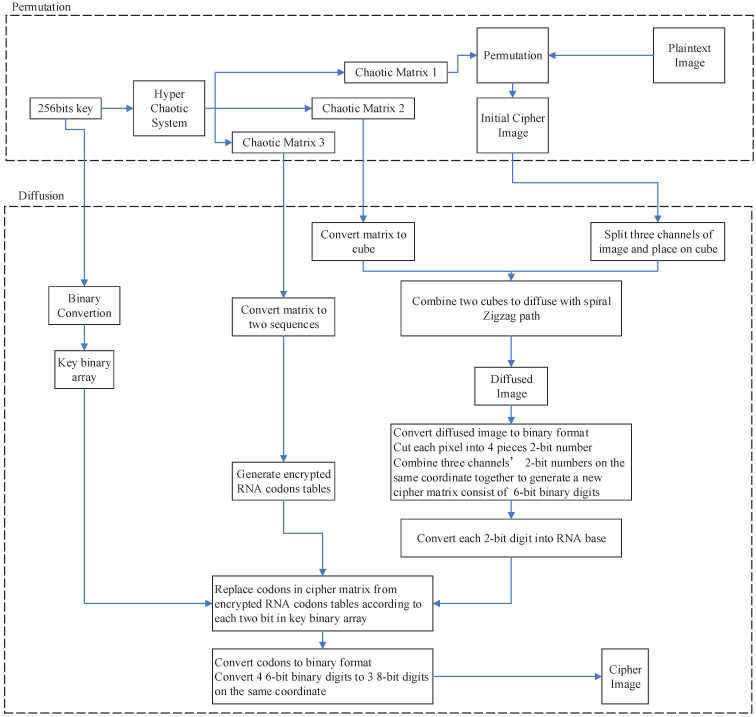
The process of encryption.

The flowchart of HCZRNA is described in Figure 6, and the specific operations are listed, as follows.

#### 3.1.1. Initial Values Generation

The HCZRNA scheme uses a 256-bit key of different characters against attacks. The 256-bit long security key would be utilized in two parts, which are hyper-chaotic system initial values generation and RNA encryption.

At first, the initial values of hyper-chaotic system should be generated by a security key. Details of initial values generation is performed in three steps:

- Step 1: divide the secret key *K* into 32 blocks, which could be expressed as $K=\{k_{1},k_{2},\cdots ,k_{32}\}$, each *k* is a 8-bits number.
- Step 2: *K* array that is generated in step 1 is calculated into four intermediate parameters $d_{1}$, $d_{2}$, $d_{3}$, $d_{4}$ by Equation (2) with four user-defined constants $c_{1}$, $c_{2}$, $c_{3}$ and $c_{4}$.
  (2) $$\left\{\begin{array}{cc} d_{1} & =c_{1}+\frac{k_{1}⊕k_{2}⊕\cdots ⊕k_{8}}{256}\\ d_{2} & =c_{2}+\frac{k_{9}⊕k_{10}⊕\cdots ⊕k_{16}}{256}\\ d_{3} & =c_{3}+\frac{k_{17}⊕k_{18}⊕\cdots ⊕k_{24}}{256}\\ d_{4} & =c_{4}+\frac{k_{25}⊕k_{26}⊕\cdots ⊕k_{32}}{256} \end{array}\right.$$
  where ⊕ represents bitwise XOR operation.
- Step 3: The initial values $x_{1}$ to $x_{6}$ of 6D hyper-chaotic system could be obtained from the 4 intermediate parameters by Equation (3).
  (3) $$\left\{\begin{array}{cc} x_{1} & =\frac{(\left(d_{1}+d_{2}\right)\times 10^{8})\;mod\;256}{255}\\ x_{2} & =\frac{(\left(d_{2}+d_{3}\right)\times 10^{8})\;mod\;256}{255}\\ x_{3} & =\frac{(\left(d_{3}+d_{4}\right)\times 10^{8})\;mod\;256}{255}\\ x_{4} & =\frac{(\left(d_{1}+d_{3}\right)\times 10^{8})\;mod\;256}{255}\\ x_{5} & =\frac{(\left(d_{1}+d_{4}\right)\times 10^{8})\;mod\;256}{255}\\ x_{6} & =\frac{(\left(d_{2}+d_{4}\right)\times 10^{8})\;mod\;256}{255} \end{array}\right.$$
  where mod means module operation.

#### 3.1.2. Hyper-Chaotic Matrices Generation

With the initial values that are calculated in Section 3.1.1, chaotic matrices could be generated from 6D hyper-chaotic system. In HCZRNA, chaotic matrices would be utilized in three parts, which are permutation, 3D transformed Zigzag diffusion, and RNA operation. Suppose that the plaintext image has N×N×3 pixels, an N×N×6 chaotic matrix is needed for permutation, a 2×N×N×6 chaotic matrix for Zigzag, and 64×6 chaotic matrix for RNA.

Therefore, the 6D hyper-chaotic system utilizes initial values from Equation (3) to iterate for generating a (3×N×N+64)×6 matrix. Given that $i^{th}$ iteration’s state values could be described as $s^{i}=\left\{x_{1,i},x_{2,i},x_{3,i},x_{4,i},x_{5,i},x_{6,i}\right\}$, a hyper-chaotic matrix *S* could be depicted as Equation (4) after all iterations.

(4) $$S=\left\{s^{1},s^{2},\cdots ,s^{M}\right\}={\left\{\begin{array}{c} x_{1,1},x_{1,2},\cdots ,x_{1,M}\\ x_{2,1},x_{2,2},\cdots ,x_{2,M}\\ x_{3,1},x_{3,2},\cdots ,x_{3,M}\\ x_{4,1},x_{4,2},\cdots ,x_{4,M}\\ x_{5,1},x_{5,2},\cdots ,x_{5,M}\\ x_{6,1},x_{6,2},\cdots ,x_{6,M} \end{array}\right\}}_{6\times M}$$

where M=3×N×N+64.

However, the numbers in matrix *S* are double-precision values, which are suitable for permutation but not for Zigzag and RNA, and color image only has three channels that are smaller than channels of *S*. Hence, matrix *S* should be separated into three pieces respectively.

For permutation, a matrix $S_{1}$ is calculated from the first N×N part of *S* by Equation (5).

(5) $$S_{1}={\left\{\begin{array}{c} x_{1,1}+x_{2,1},x_{1,2}+x_{2,2},\cdots ,x_{1,M^{\prime }}+x_{2,M^{\prime }}\\ x_{3,1}+x_{4,1},x_{3,2}+x_{4,2},\cdots ,x_{3,M^{\prime }}+x_{4,M^{\prime }}\\ x_{5,1}+x_{6,1},x_{5,2}+x_{6,2},\cdots ,x_{5,M^{\prime }}+x_{6,M^{\prime }} \end{array}\right\}}_{3\times M^{\prime }}$$

where $M^{\prime }=N\times N$.

While matrix $S_{2}$ is cut from $s^{M^{\prime }+1}$ to $s^{3M^{\prime }}$ in *S* for 3D transformed Zigzag diffusion. Additionally, because 8-bit integer digits are needed for diffusion, each item $x_{i,j}^{\prime }$ in $S_{2}$ should be calculated by Equation (6).

(6) $$\begin{array}{cc} Suppose\;\vec{s_{i}} & =\{x_{i,(M^{\prime }+1)},x_{i,(M^{\prime }+2)},\cdots ,x_{i,3M^{\prime }}\}\\ y_{i,j} & =2\times x_{i,j}+\frac{max\left(\vec{s_{i}}\right)+min\left(\vec{s_{i}}\right)}{max\left(\vec{s_{i}}\right)-min\left(\vec{s_{i}}\right)}\\ x_{i,j}^{\prime } & =\left((\lfloor \right|y_{i,j}\left|-\lfloor \right|y_{i,j}\left|\rfloor \rfloor \right)\times 10^{10})\;mod\;256 \end{array}$$

where max and min are maximum and minimum operations.

Matrix $S_{3}$ is the last part of matrix *S* and it is used to sort operation for encrypting RNA codons tables as indexes. Because there only needs two indexes sequences, matrix $S_{3}$ should be summarized as Equation (7).

(7) $$\begin{array}{c} index_{1}=\left\{x_{1,3M^{\prime }+1}+x_{2,3M^{\prime }+1}+x_{3,3M^{\prime }+1},x_{1,3M^{\prime }+2}+x_{2,3M^{\prime }+2}+x_{3,3M^{\prime }+2},\cdots ,x_{1,3M^{\prime }+64}+x_{2,3M^{\prime }+64}+x_{3,3M^{\prime }+64}\right\}\\ index_{2}=\left\{x_{4,3M^{\prime }+1}+x_{5,3M^{\prime }+1}+x_{6,3M^{\prime }+1},x_{4,3M^{\prime }+2}+x_{5,3M^{\prime }+2}+x_{6,3M^{\prime }+2},\cdots ,x_{4,3M^{\prime }+64}+x_{5,3M^{\prime }+64}+x_{6,3M^{\prime }+64}\right\} \end{array}$$

#### 3.1.3. Permutation

In this part, matrix $S_{1}$ is used to permute plaintext image. At the beginning, each element in $S_{1}$ should be allocated to each pixel as index. Hence, an N×N×3 matrix $S_{1}^{\prime }$ is needed to be converted from $S_{1}$ by reshaping.

(8) $$S_{1}^{\prime }=reshape\left(S_{1},N,N,3\right)$$

Afterwards, each pixel in plaintext image has a corresponding index in $S_{1}^{\prime }$ at the same coordinate. Combine plaintext image with matrix $S_{1}^{\prime }$, and take another reshaping operation to convert these two matrix into two sequences with a length of N×N×3. After sorting $S_{1}^{\prime }$ ascendingly with image sequence synchronously, pixels’ orders in plaintext image sequence have been scrambled.

Finally, reshaping the sorted image sequence to an N×N×3 matrix, the initial cipher image could be generated.

#### 3.1.4. Diffusion

After permutation, a diffusion scheme by 3D Zigzag transformation is proposed, as follows. An initial cipher image would be split and placed on the surfaces of an N×N×6 cube, termed as *P*, as described in Section 2.2. Additionally, chaotic matrix $S_{2}$ would also be placed on another two N×N×6 cubes, since diffusion would implement two rounds. For the first N×N×6 numbers in $S_{2}$, each number would be placed on a cube in order, which could be called cube $SC_{1}$.For the last N×N×6 numbers in $S_{2}$, cube $SC_{2}$ could be generated by the same process.

Subsequently, diffusion would start from origin point of cube *P* on the front side, and its coordinate is [1,1,1]. At each iteration, the pixel’s value $C_{i,j,m}$ is calculated by Equation (9).

(9) $$C_{i,j,m}=\left(P_{i,j,m}⊕\left(T+x_{i,j,m;1}\right)\right)\;mod\;256$$

where i,j,m are the coordinates of pixel at the $i^{th}$ row, $j^{th}$ column, and $m^{th}$ side on the cube. *T* is the previous one diffused pixel’s *C* value, if i,j,m=1,1,1, *T* is a user-defined constant. $x_{i,j,m;1}$ is the corresponding coordinate’s value in $SC_{1}$.

For the second round of diffusion, Equation (9) would change to Equation (10).

(10) $$D_{i,j,m}=\left(C_{i,j,m}⊕\left(T^{\prime }+x_{i,j,m;2}\right)\right)\;mod\;256$$

where D is result of diffusion, and $T^{\prime }$ is the previous one diffused pixel’s *D* value, and, if i,j,m=1,1,1, $T^{\prime }$ is the last pixel’s *C* value after the first round diffusion. While $x_{i,j,m;2}$ is corresponding coordinate’s value in $SC_{2}$.

Through these two round diffusions, *D* cube is generated. Additionally, recover the D’s N×N×6 matrix by reversing processes of image splitting and cube placement in Section 2.2. A diffused N×N×3 matrix $D_{mat}$ is obtained.

#### 3.1.5. RNA Operation

The encryption from diffused matrix $D_{mat}$ through RNA operation could be described, as follows:

- Step 1: RNA operation is initiated from creating two encrypted codons tables, called $T_{00}$ and $T_{01}$. In which, $T_{00}$ and $T_{01}$ are shuffled tables from codons truth, as in Table 2. The shuffle orders are generated according to indexes sequences calculated from Equation (7). After sorting with these two indexes sequences, the original codons truth table could be shuffled to two different encrypted codons tables $T_{00}$ and $T_{01}$.
  Subsequently, by the complementary rules of RNA, additional tables $T_{10}$ and $T_{11}$ could be generated from $T_{00}$ and $T_{01}$. Hence, four encrypted codons tables are generated.
- Step 2: for each element in $D_{mat}$, binary number conversion is processed, which is recorded as *B*.
  (11) $$B=\{b_{i,j,m}\}.\;i,j=1,2,\cdots ,N;m=1,2,3$$
  Each $b_{i,j,m}$ could be expressed as eight binary numbers, which could be depicted as $b_{0}^{i,j,m}b_{1}^{i,j,m}b_{2}^{i,j,m}b_{3}^{i,j,m}b_{4}^{i,j,m}b_{5}^{i,j,m}b_{6}^{i,j,m}b_{7}^{i,j,m}$.
- Step 3: divide $b_{i,j,m}$ into four pieces, each two bits are one piece, which are recorded as:
  (12) $$\begin{array}{c} bt_{1}^{i,j,m}=b_{0}^{i,j,m}b_{1}^{i,j,m}\\ bt_{2}^{i,j,m}=b_{2}^{i,j,m}b_{3}^{i,j,m}\\ bt_{3}^{i,j,m}=b_{4}^{i,j,m}b_{5}^{i,j,m}\\ bt_{4}^{i,j,m}=b_{6}^{i,j,m}b_{7}^{i,j,m} \end{array}$$
  Additionally, combine three channels’ bts at the same coordinate together:
  (13) $$\begin{array}{c} bt_{1}^{i,j}=bt_{1}^{i,j,1}bt_{1}^{i,j,2}bt_{1}^{i,j,3}\\ bt_{2}^{i,j}=bt_{2}^{i,j,1}bt_{2}^{i,j,2}bt_{2}^{i,j,3}\\ bt_{3}^{i,j}=bt_{3}^{i,j,1}bt_{3}^{i,j,2}bt_{3}^{i,j,3}\\ bt_{4}^{i,j}=bt_{4}^{i,j,1}bt_{4}^{i,j,2}bt_{4}^{i,j,3} \end{array}$$
  Therefore, each $bt^{i,j}$ has six bits that could transfer to RNA codons according to Table 1. Exchange each two bits in bts to RNA base one-by-one according to the principle of row priority, bts could be coded to codons. And put them into a one-dimension sequence BS as Equation (14).
  (14) $$BS=\{bt_{1}^{1,1},bt_{2}^{1,1},bt_{3}^{1,1},bt_{4}^{1,1},bt_{1}^{1,2},bt_{2}^{1,2},\cdots ,bt_{4}^{2,1},bt_{1}^{2,2},\cdots ,bt_{3}^{N,N},bt_{4}^{N,N}\}.$$
- Step 4: convert key to binary format. 256-bit key could be changed into a binary sequence BK.
  (15) $$\begin{array}{cc} key & =[key_{0},key_{1},\cdots ,key_{31}]\\ key_{i} & =key_{i,0},key_{i,1},\cdots ,key_{i,7}\\ BK & =[key_{1,0},key_{1,1},\cdots ,key_{1,7},key_{2,0},key_{2,1},\cdots ,key_{31,7}] \end{array}$$
  Walk through sequence BS, and find corresponding index id of each codon in BS from Table 2. For each codon in BS, check 2-bits table number *z* in sequence BK.
  (16) $$z=BK_{n\;mod\;2048}BK_{(n+1)\;mod\;2048}$$
  where *n* is the walking times.
  Take the codon $T_{z}\left(id\right)$ to replace the origin codon BS(n).
  When iterations termination, an encrypted sequence is generated.
- Step 5: decode each base in encrypted sequence BS to binary format by Table 1, put all of the binary digits back to original coordinates by reversing operations in Step 3. Additionally, change binary matrix into 2-bit matrix. The cipher image is generated.

The HCZRNA encryption has four stages: hyper-chaotic matrices generation (Section 3.1.1 and Section 3.1.2), hyper-chaotic permutation (Section 3.1.3), 3D transformed Zigzag diffusion on surfaces of cubes, which is generated from initial cipher image (Section 3.1.4), and a bit-level RNA operation (Section 3.1.5). The major steps of the HCZRNA are Section 3.1.4 and Section 3.1.5, i.e., the transformed Zigzag diffusion on 3D cubes and bit-level RNA substitutions with hyper-chaotic matrix, respectively. The HCZRNA uses the strategy of “divide and conquer” that is widely used in various applications to decompose the original encryption task into a couple of simpler sub-tasks [38,39].

### 3.2. Decryption

In this paper, the encryption scheme has been depicted, and decryption is the inverse process of encryption. Details are proposed, as follows.

**Figure 7:**
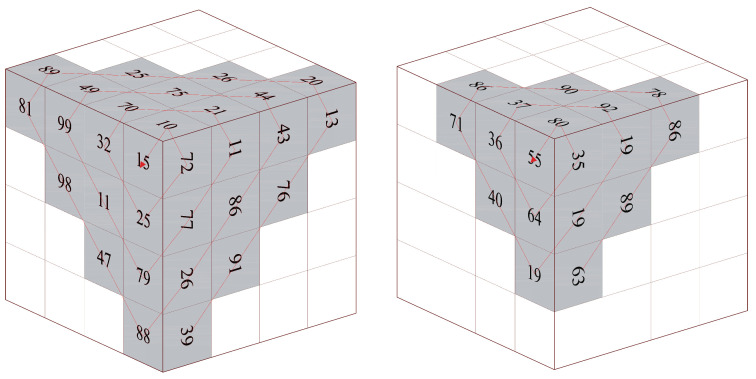
Reverse traversal.

**Figure 8:**
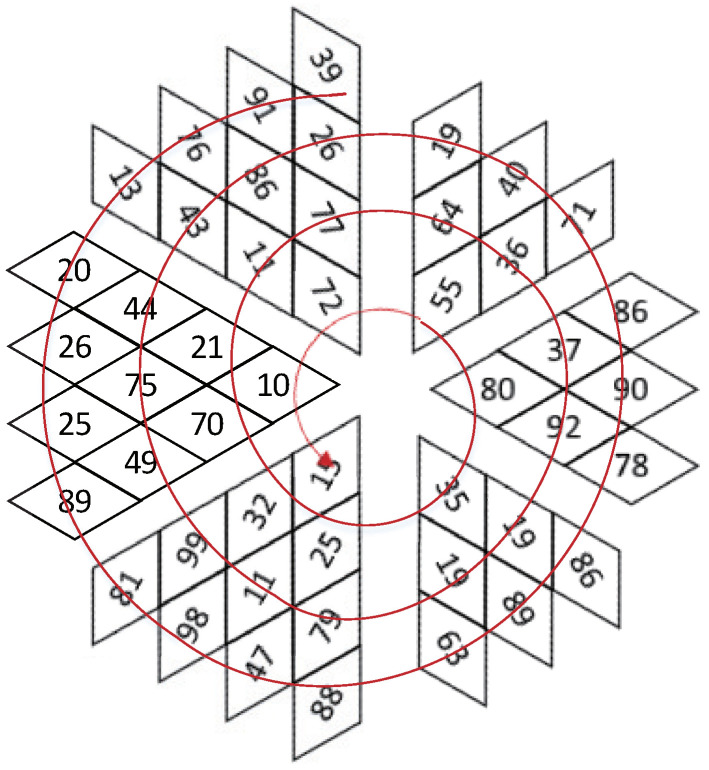
The order of Zigzag in decryption.

- Step 1: redo the processes that are listed in Section 3.1.1 and Section 3.1.2 to generate hyper-chaotic matrices $S_{1}$, $S_{2}$, and $S_{3}$.
- Step 2: convert the cipher image to a binary format, and reconstruct three channels’ pixels at each coordinate into four 6-bit binary arrays by using Euqation (12) and (13). Change 6-bit arrays into codons from codons truth Table 2, and put them in a one-dimension sequence $BS^{\prime }$ as Equation (14).
- Step 3: generate key binary sequence BK through Equation (15) and encrypted codons tables $\left\{T_{00},T_{01},T_{10},T_{11}\right\}$ by redoing Step 1 in Section 3.1.5.
- Step 4: Check each 2-bits *z* in BK and find corresponding table $T_{z}$ from $\left\{T_{00},T_{01},T_{10},T_{11}\right\}$. Walk through $BS^{\prime }$ and find each codon’s corresponding index $id^{\prime }$ in Table $T_{z}$. Replace codon in $BS^{\prime }$ to codon $id^{\prime }$ in codons truth Table 2. After all codons are replaced, convert them into binary formats and 8-bit numbers, matrix $D_{mat}^{\prime }$ is obtained.
- Step 5: split matrix $D_{mat}^{\prime }$ and place triangles on cube surfaces as the process shown in Section 2.2. Redo Section 3.1.4 with modified Equation (17) two rounds, and then walk through pixels with reversed Zigzag path. Take Figure 4 in Section 2.2 as an example, the traversal road of decryption is shown in Figure 7. If we put all the pixels together, the order of traversal is depicted in Figure 8.
  (17) $$\begin{array}{cc} C_{i,j,m}^{\prime } & =(D_{i,j,m}^{\prime }⊕\left(T^{\prime }+x_{i,j,m;2}\right))mod256\\ P_{i,j,m}^{\prime } & =(C_{i,j,m}^{\prime }⊕\left(T+x_{i,j,m;1}\right))mod256 \end{array}$$
  where $T^{\prime }$ is the previous one pixel’s $D^{\prime }$ value, and, if i,j,m=1,1,1, $T^{\prime }$ is the last pixel’s $C^{\prime }$ value after first round iteration. *T* is the previous one pixel’s $C^{\prime }$ value and, if i,j,m=1,1,1, *T* is the user-defined constant that is used in Section 3.1.4.
- Step 6: after the process in Step 5, return the triangles in the cube to theirs original coordinates on a image. Additionally, the reverse processes in Section 3.1.3, reshape $S_{1}$ to construct sorted sequence. Find image pixels’ corresponding coordinates through sorted sequence and recover. The decrypted image is generated.

## 4. Experimental Results

The encryption and decryption schemes have been tested on four popular RGB color images in Table 3. All of the experiments are conducted by MATLAB R2019b on 64-bit Windows 10 system, and the main hardware includes an Xeon(R) W-2223 @ 3.60 GHz CPU as well as 32 GB RAM.

For the controlling parameters setting in Equation (1), (a,b,c,d,e,f,g,r,ω,β)=(0.3,1.5,8.5,−2,1,−0.1,0.9,1,1,0.2). Constants $c_{1},c_{2},c_{3},c_{4}$ in Equation (2) are set as (1,1,2,2) and the initial constant of *T* in Equation (9) is 11. The security key can be set by users, so we set a 256-bit hexadecimal sequence that is shown below as the security key in all of the experiments. The key can also be optimized by some evolutionary optimizations, such as differential evolution and particle swarm optimization [40,41,42,43].

$$key{=}^{\prime }743B5A203B1E8EDF6C0FB0D7497CB2E228689AD00F57F8953B5C6127E1C26053^{\prime }$$

In order to demonstrate performance of proposed HCZRNA scheme, five state-of-the-art encryption schemes are employed for comparison: a Four-wing hyper-chaotic system based dynamic DNA encryption scheme [29], an extended Zigzag confusion and RNA encryption based scheme [23], a Hopfield chaotic neural network-based scheme [44], a scheme with utilization of differences between two 1D chaotic maps [45] and a scheme with 4D hyper-chaotic system and DNA encryption [46].

### 4.1. Key Space

For an image encryption system, large enough key space is necessary to withstand a brute-force attack. In HCZRNA, a 256-bit security key is used to calculate the initial values of the hyper-chaotic system to generate the pseudo random matrices that could affect the outputs of permutation, diffusion, and RNA operations. As we know, different initial values in a hyper-chaotic system would get different pseudo random sequences, and each bit has two states, the security key has $2^{256}$ different states, so it could generate $2^{256}$ results of a hyper-chaotic system. Therefore, the key space of HCZRNA could be calculated as $2^{256}$. Theoretically, if the key space of an encryption scheme is larger than $2^{100}$, this scheme could resist violent crack by modern computers [47]. Therefore, the proposed HCZRNA in this paper has a large enough key space to resist brute-force attack.

### 4.2. Sensitivity of Keys

The sensitivity test on keys refers to utilize slightly different keys to encrypt the same images. If an encryption is sensitive, the encryption with slight difference on keys would get completely different cipher images. To test the key sensitivity, we would use two different keys to encrypt four test images, one of these two keys is initial security key $key_{1}$, another key is $key_{2}$, which is one bit changed for $key_{1}$. These two keys are stated as follows, where the changed bits are shown in red:

$$\begin{array}{cc} key_{1} & {=}^{\prime }743B5A203B1E8EDF6C0FB0D7497CB2E228689AD00F57F8953B5C6127E1C26053^{\prime }\\ key_{2} & {=}^{\prime }743B5A203B1F8EDF6C0FB0D7497CB2E228689AD00F57F8953B5C6127E1C26053^{\prime } \end{array}$$

By comparing two cipher images from the same plaintext image, the differences of cipher images that are encrypted from these two security keys are stated in Table 4.

In the table, it is obvious that all of the differences between two cipher images are over 99%, which reveals that, even with tiny changes in security keys, encryption by HCZRNA would also lead to extremely different outputs. Hence, HCZRNA satisfies sensitivity requirements.

### 4.3. Histogram

**Figure 9:**
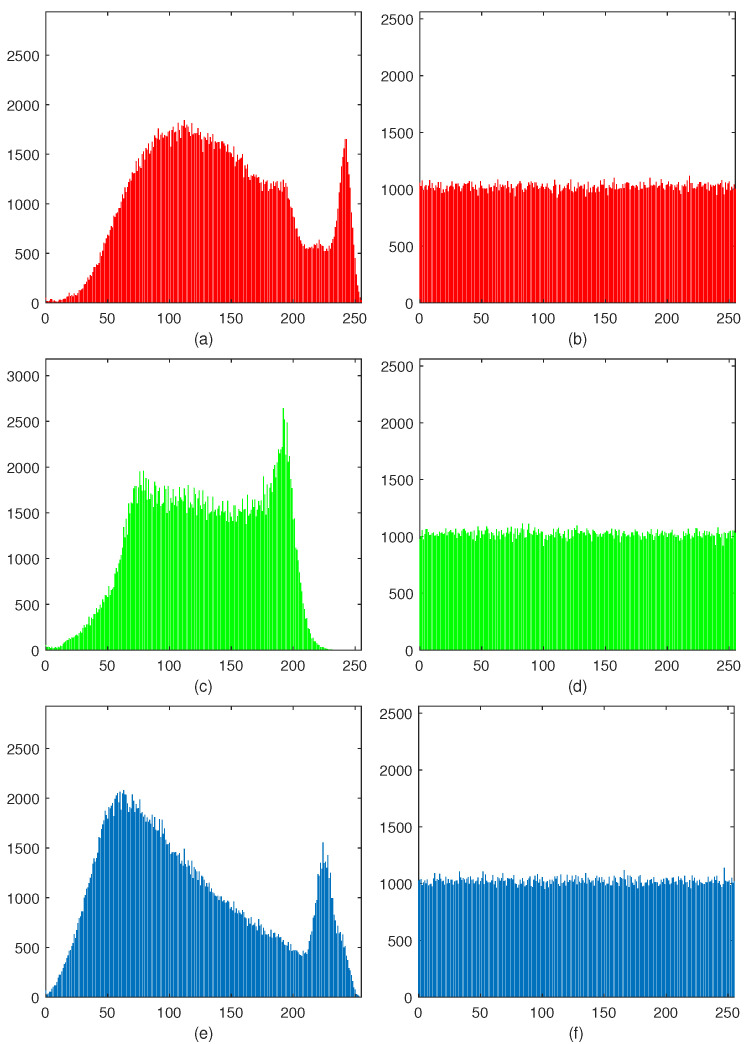
Histogram. image (**a**,**c**,**e**) are the histograms of three channels of Baboon, and image (**b**,**d**,**f**) are the histograms of corresponding channels of encrypted Baboon.

Because a histogram reflects each pixel’s times in an image, histograms of meaningful images are fluctuated, while cipher images’ histogram should be flat and uniform. That is to say, if an encryption scheme is well-designed, the histograms of cipher images should be as flat as possible. For the proposed HCZRNA, a histogram of Baboon and its cipher image are placed in Figure 9.

From this figure, it could find that histograms of all channels in plaintext image are fluctuated, while histograms of cipher image’s different channels are almost distributed in a narrow range, and their values are around 1000. For more accurate results, histogram statistics are introduced to evaluate the variance and standard deviation of plaintext and cipher images [48,49]. Variance is used to calculate the average difference in each gray level frequency with respect to mean value $\bar{x}$, which could be formulated as Equation (18).

(18) $$\begin{array}{cc} \alpha & =\frac{1}{256}\sum_{i=1}^{256}{\left(x_{i}-\bar{x}\right)}^{2},\\ \bar{x} & =\frac{h\times w}{256} \end{array}$$

where h,w represent the image’s height and width respectively, *x* is the frequency of different gray levels of pixels in a image, and the $\bar{x}$ is the mean value of *x*s. And α is the variance, the higher is α, the more fluctuate is the graphic histogram. Accordingly, if a encryption is well-designed, the α of encrypted image should be low.

As α is always very high in plaintext image, a standard deviation is used to evaluate histogram’s fluctuations, which is stated as Equation (19).

(19) $$\beta =\sqrt{\alpha }$$

where β is the standard deviation. For all test images, Table 5 describes the results of histogram statistics.

In the table, the variances and standard deviations of plaintext images are very high, while they are extremely different in cipher images. All of these performances indicate that the proposed HCZRNA could effectively resist histogram attack.

### 4.4. Correlation

The correlation test refers to adjacent pixels’ relationship. A meaningful image has high correlation because values of adjacent pixels are close to each other. This attribute could be utilized to crack. Therefore, a well-designed encryption scheme should have low enough correlations in three directions: horizontal, vertical, and diagonal directions. Given a pixel sequence that is represented by $X=\{x_{1},x_{2},\cdots ,x_{N}\}$ and its adjacent pixel sequence $Y=\{y_{1},y_{2},\cdots ,y_{N}\}$ in an image, correlation between *X* and *Y* could be denoted as $\gamma_{X,Y}$ in Equation (20).

(20) $$\begin{array}{cc} \gamma_{X,Y} & =\frac{\frac{1}{N}\sum_{i=1}^{N}\left(x_{i}-D\left(X\right)\right)\left(y_{i}-D\left(Y\right)\right)}{\sqrt{D(X)D(Y)}}\\ D(X) & =\frac{\sum_{i=1}^{N}{\left(x_{i}-E\left(X\right)\right)}^{2}}{N}\\ E(X) & =\frac{\sum_{i=1}^{N}x_{i}}{N} \end{array}$$

where E(X) is X’s mathematical expectation and D(X) is standard deviation.

If *X* and *Y* are identical, $\gamma_{X,Y}$ would be a maximum of 1. On the contrary, $\gamma_{X,Y}$ would be close to 0 when *X* and *Y* have few correlations.

**Figure 10:**
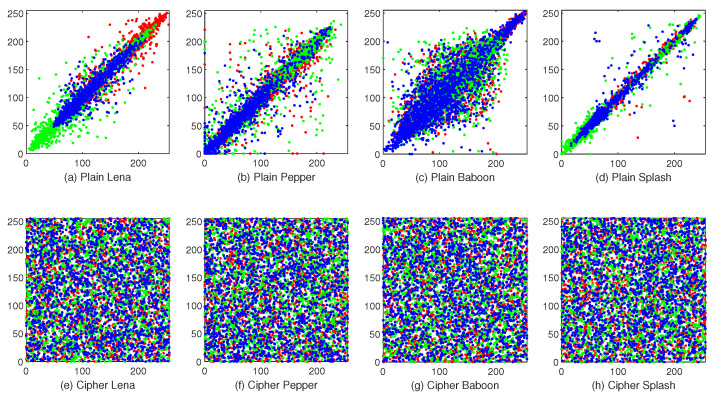
Correlations.The first row is correlations of plaintext images, and the second row is correlations of cipher images.

Figure 10 depicts the correlation test results. It is obvious that the adjacent pixels’ distributions in plaintext images are concentrated, while the distributions in the cipher images are opposite.

More accurately, Table 6 provides correlation coefficients between plaintext images and cipher images. Additionally Table 7 demonstrates comparisons with references [44,45]. Through this test, it could find that the correlation coefficients of the proposed HCZRNA are extremely close to 0, which means that HCZRNA could effectively break correlations existing in plaintext images. While the comparisons show that the proposed HCZRNA achieves the best results with [44,45] in all cases. This reveals that HCZRNA outperforms when compared schemes in terms of reducing correlations.

### 4.5. Information Entropy

Information Entropy shows the randomness and uncertainty of image’s pixels. If pixels in an image have uniform distribution, this image could resistant statistical attacks. Because there are 256 gray levels in each channel of color image, the Entropy calculation could be formulated as Equation (21):

(21) $$H\left(C\right)=-\sum_{i=0}^{255}p\left(i\right)log_{2}p\left(i\right)$$

where *C* denotes channels of color image and p(i) is probability of gray level in whole channel.

The bigger H(C), the bigger uncertainty of image. While the theoretical value of H(C) is 8.

Table 8 shows the entropies of all channels of plaintext color images and corresponding cipher images through encryptions by proposed HCZRNA. It is obvious that cipher images have increased entropies a lot from plaintext images and their entropies are very close to the theoretical value. Moreover, a comparison is held between HCZRNA and Refs. [23,29,44,45,46], and the results are stated in Table 9. Among all of the encryption schemes, the proposed HCZRNA achieves the highest entropies in four out of six cases. It could conclude that HCZRNA has the ability to resist statistical attack.

### 4.6. Differential Attack

The differential attack test is an important security test for image encryption, which reveals the influence on the cipher image caused by a minor change in pixels of plaintext image. If a tiny change on pixels in plaintext image leads to significant different cipher image, that is to say the encryption scheme could resist differential attack.

Two important indices are introduced to measure the ability of differential attack resistance, which is called the number of pixel change rate (NPCR) and the unified average changing intensity (UACI). Additionally, they are defined as Equations (22) and (23):

(22) $$NPCR=\frac{\sum_{i=0}^{h}\sum_{j=0}^{w}F\left(i,j\right)\times 100\%}{w\times h}$$

(23) $$UACI=\frac{\sum_{i=0}^{h}\sum_{j=0}^{w}\left|e_{1}\left(i,j\right)-e_{2}\left(i,j\right)\right|}{255\times w\times h}$$

where $e_{1}$ and $e_{2}$ are two cipher images, and e(i,j) means the pixel’s value at coordinate i,j in image *e*. F(i,j) denotes whether the same coordinate’s pixel values in $e_{1}$ and $e_{2}$ are independent or not, which could be formulated as Equation (24):

(24) $$F\left(i,j\right)=\left\{\begin{array}{c} 0,\;if\;e_{1}\left(i,j\right)=e_{2}\left(i,j\right)\\ 1,\;if\;e_{1}\left(i,j\right)\neq e_{2}\left(i,j\right) \end{array}\right.$$

For two random images, NPCR and UACI’s expected values are stated as: NPCR=99.6094% and UACI=33.4635% for an 8-bit gray image [30].

Hence, to realize the test, one bit would be changed on a random pixel in plaintext image. And both the plaintext image and changed image are encrypted to two different cipher images. Table 10 lists the average results of ten times tests. It could find that all NPCR values and UACI values of cipher images’ different channels exceed the theoretical values. Additionally, comparisons with Refs. [23,29,44,45,46] are shown in Table 11 and Table 12. Through the comparisons, the proposed HCZRNA encryption scheme has better performances on NPCR and UACI, which indicates that HCZRNA could resist differential attack well.

### 4.7. Robustness

It is unavoidable that there data loss or noise attack occur when cipher images are transmitting. Hence, a well-designed encryption and decryption scheme should resist contamination on cipher images to recover plaintext images without great changes.

**Figure 11:**
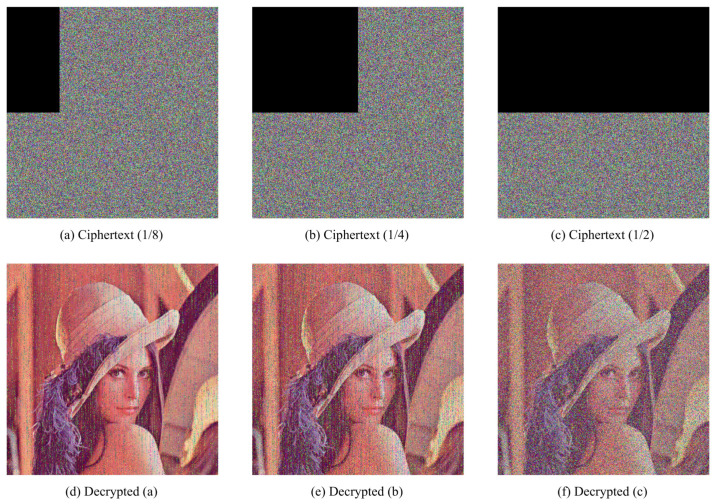
Cropping attack tests. The first row is cipher images with 12.5%, 25% and 50% data loss, and the second row is decrypted images from the first row.

**Figure 12:**
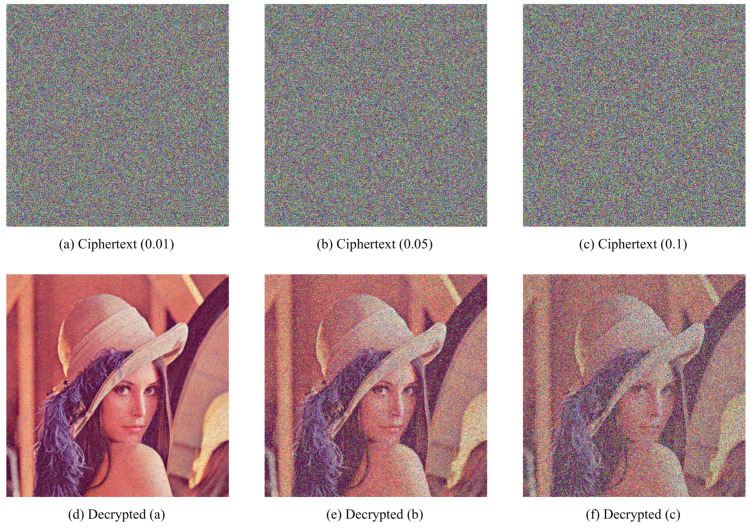
Noise attack tests. The first row is cipher images with 1%, 5%, 10% salt and pepper noise, and the second row is decrypted images from the first row.

To demonstrate robustness of proposed HCZRNA scheme, 12.5%, 25%, and 50% data lose tests and 1%, 5%, and 10% salt and pepper noise tests would presented in Figure 11 and Figure 12.

From the figures, the main information of plaintext images could be identified from decrypted images, which could conclude that HCZRNA has enough robustness for data loss and noise attacks. Here, the Mean Squared Error (MSE) and Peak Signal to Noise Ratio (PSNR) are also utilized to test robustness [48,49], which is formulated as Equation (25).

(25) $$\begin{array}{cc} MSE & =\frac{1}{h\times w}\sum_{i=1}^{h}\sum_{j=1}^{w}{\left[P\left(i,j\right)-E\left(i,j\right)\right]}^{2},\\ PSNR & =20log_{10}\left(\frac{255}{\sqrt{MSE}}\right) \end{array}$$

where *P* and *E* represent two different images. MSE is used to evaluate the difference between two images, and PSNR depicts the ratio between the maximum possible power of a signal and the power of distorting noise that affects the quality of its representation. The lower the MSE, the higher PSNR, which indicates that two images have high similarity. Hence, under noise attacks, if the PSNR between the plaintext image and decrypted image is high, the encryption and decryption schemes are good enough. Table 13 and Table 14 present the results of plaintext image and decrypted image of Lena under data loss and salt and pepper noise attacks.

Through the results, we could find there are high MSEs and low PSNRs in these tables, which figures out that HCZRNA could resist attacks of data loss and noise.

### 4.8. Running Time

In HCZRNA, the pixels of image would be walked through multiple times in diffusion and RNA operation. Suppose that the size of RGB image is N×N×3. For the five parts of encryption processes that are listed in Section 3.1, initial values of hyper-chaotic system are calculated from the security key, which costs O(1) time complexity; the hyper-chaotic matrices are computed 3×N×N+64 times iterations; for permutation, reshape and sort operations are implemented three times; while the diffusion process walks through each pixel two times, which costs O(2×N×N×3); at last, as RNA operation walks through all 6-bit codons that are transformed from 8-bit pixels, the times of iteration are increased to $\frac{4}{3}\times N\times N\times 3$. Hence, the time complexity of HCZRNA could be calculated as $O\left(1+3\times N\times N+64+3+2\times N\times N\times 3+\frac{4}{3}\times N\times N\times 3\right)=O\left(13N^{2}+68\right)=O\left(N^{2}\right)$. Using the experiment environment that is listed in this section, the running times of encryption and decryption could be stated in Table 15. Although the time costs of encryption and decryption are not very good, the time complexity is also a polynomial time, which could be tolerable. Additionally, the processes of RNA operation on different codons have no correlation with each other, which could improve computational time by computing RNA operation in parallel.

## 5. Conclusions

A novel hyper-chaotic system based image encryption scheme is proposed with 3D transformed Zigzag and RNA operation in this paper. By using the 6D hyper-chaotic system, three auxiliary matrices are generated, including one permutation index matrix, one mask matrix for Zigzag, and one codon table index matrix. Subsequently, two rounds 3D transformed Zigzag diffusion mechanism is proposed for pixels diffusion with each other. Nevertheless, additional encryption with RNA codons makes more reliable and secure results through employing codons tables and security keys. Through simulations, the proposed HCZRNA has better performances on the resistance of different types attacks than the compared encryption schemes, while the speed is not ideal, since it is a complex process. On the premise of ensuring performance, we would simplify diffusion and RNA operation processes and optimize the encryption steps for improving speed in the future. 

## Figures and Tables

**Table 1:** Binary representation of RNA.

RNA Bases	A	C	G	U
Binary	00	01	10	11

**Table 2:** RNA codon table.

#	Bin.	Codon	#	Bin.	Codon	#	Bin.	Codon	#	Bin.	Codon
0	000000	AAA	16	010000	CAA	32	100000	GAA	48	110000	UAA
1	000001	AAC	17	010001	CAC	33	100001	GAC	49	110001	UAC
2	000010	AAG	18	010010	CAG	34	100010	GAG	50	110010	UAG
3	000011	AAU	19	010011	CAU	35	100011	GAU	51	110011	UAU
4	000100	ACA	20	010100	CCA	36	100100	GCA	52	110100	UCA
5	000101	ACC	21	010101	CCC	37	100101	GCC	53	110101	UCC
6	000110	ACG	22	010110	CCG	38	100110	GCG	54	110110	UCG
7	000111	ACU	23	010111	CCU	39	100111	GCU	55	110111	UCU
8	001000	AGA	24	011000	CGA	40	101000	GGA	56	111000	UGA
9	001001	AGC	25	011001	CGC	41	101001	GGC	57	111001	UGC
10	001010	AGG	26	011010	CGG	42	101010	GGG	58	111010	UGG
11	001011	AGU	27	011011	CGU	43	101011	GGU	59	111011	UGU
12	001100	AUA	28	011100	CUA	44	101100	GUA	60	111100	UUA
13	001101	AUC	29	011101	CUC	45	101101	GUC	61	111101	UUC
14	001110	AUG	30	011110	CUG	46	101110	GUG	62	111110	UUG
15	001111	AUU	31	011111	CUU	47	101111	GUU	63	111111	UUU

**Table 3:** Testing images.

Image	Size (h×w×c)	Image	Size (h×w×c)
Lena	256×256×3	Baboon	512×512×3
Peppers	256×256×3	Splash	512×512×3

**Table 4:** Differences between the cipher images.

Image	Lena	Pepper	Baboon	Splash
Difference	99.59%	99.62%	99.61%	99.60%

**Table 5:** Histogram statistics.

Image	Channels	Plaintext		Ciphertext	
		α	β	α	β
Lena	R	65,306	255	248	15
	G	30,665	175	258	16
	B	91,939	303	232	15
Pepper	R	57,413	239	249	15
	G	119,411	345	238	15
	B	151,644	389	237	15
Baboon	R	165,679	407	520	22
	G	285,616	534	532	23
	B	159,885	399	541	23
Splash	R	1,211,325	1100	566	23
	G	1,541,948	1241	495	22
	B	2,958,482	1720	504	22

**Table 6:** The correlation coefficients of the testing images.

Image	Channels	Plaintext			Ciphertext		
		Horizontal	Vertical	Diagonal	Horizontal	Vertical	Diagonal
Lena	R	0.9512	0.9755	0.9444	0.0046	0.0024	0.0051
	G	0.9512	0.9679	0.9276	−0.0027	−0.0007	0.0002
	B	0.9512	0.9479	0.9021	−0.0023	0.0014	0.0004
Baboon	R	0.9218	0.8624	0.8531	0.0003	0.0001	0.0015
	G	0.9218	0.7591	0.7299	−0.0010	0.0004	0.0020
	B	0.9218	0.8782	0.8411	0.0005	−0.0022	0.0012

**Table 7:** Comparisons of correlation coefficients.

Image	Channels		Plaintext	Ciphertext		
				HCZRNA	Ref. [44]	Ref. [45]
Baboon	R	Horizontal	0.9218	**0.0003**	0.0054	−0.0073
		Vertical	0.8624	**0.0001**	−0.0042	−0.0059
		Diagonal	0.8531	**0.0015**	−0.0177	−0.0136
	G	Horizontal	0.9218	**−0.0010**	−0.0055	0.0046
		Vertical	0.7591	**0.0004**	0.0119	−0.0077
		Diagonal	0.7299	**0.0020**	0.0046	−0.0044
	B	Horizontal	0.9218	**0.0005**	−0.0021	−0.0067
		Vertical	0.8782	**−0.0022**	0.0104	−0.0111
		Diagonal	0.8411	**0.0012**	−0.0021	0.0122

**Table 8:** Information Entropies of testing images.

Image	Channels	Lena	Peppers	Baboon	Splash
Plaintext	R	7.2353	7.3369	7.7067	6.9481
	G	7.5683	7.4394	7.4744	6.8845
	B	6.9176	7.0219	7.7522	6.1265
Ciphertext	R	7.9973	7.9972	7.9993	7.9993
	G	7.9970	7.9970	7.9993	7.9994
	B	7.9972	7.9972	7.9993	7.9993

**Table 9:** Comparison of entropies.

Image	Channel	Plaintext	HCZRNA	Ref. [29]	Ref. [23]	Ref. [44]	Ref. [45]	Ref. [46]
Lena	R	7.2353	**7.9973**	7.9971	**7.9973**	-	-	**7.9973**
	G	7.5683	7.9970	7.9971	7.9972	-	-	**7.9975**
	B	6.9176	7.9972	7.9971	7.9971	-	-	**7.9975**
Baboon	R	7.7067	**7.9993**	7.9926	-	**7.9993**	**7.9993**	7.9970
	G	7.4744	**7.9993**	7.9926	-	**7.9993**	**7.9993**	7.9978
	B	7.7522	**7.9993**	7.9926	-	**7.9993**	7.9992	7.9987

**Table 10:** The mean number of pixel change rate (NPCR) and unified average changing intensity (UACI) of cipher images.

Image	NPCR(%)			UACI(%)		
	R	G	B	R	G	B
Lena	99.6619	99.6272	99.6460	33.6177	33.6048	33.6422
Peppers	99.6481	99.6404	99.6239	33.7208	33.5701	33.6435
Baboon	99.6159	99.6769	99.6115	33.5196	33.5203	33.5049
Splash	99.6219	99.6934	99.6253	33.4983	33.5114	33.4816

**Table 11:** Average NPCR (%) of running the schemes 10 times.

Image	Channel	HCZRNA	Ref. [29]	Ref. [23]	Ref. [44]	Ref. [45]	Ref. [46]
Lena	R	**99.6619**	99.60	99.6323	-	-	99.615
	G	**99.6272**	*99.61*	99.6109	-	-	99.62
	B	**99.6460**	*99.61*	99.6338	-	-	99.617
Baboon	R	**99.6159**	99.6083	-	*99.6037*	*99.6037*	99.6140
	G	**99.6769**	99.6065	-	99.6048	*99.6017*	99.6073
	B	99.6115	99.6094	-	99.6059	*99.6043*	**99.6292**

**Table 12:** Average UACI (%) of running the schemes 10 times.

Image	Channel	HCZRNA	Ref. [29]	Ref. [23]	Ref. [44]	Ref. [45]	Ref. [46]
Lena	R	**33.6177**	33.56	*33.4683*	-	-	33.4732
	G	**33.6048**	33.45	*33.4341*	-	-	33.3428
	B	**33.6422**	33.49	33.4991	-	-	*33.4647*
Baboon	R	**33.5196**	33.4939	-	33.4427	*29.9630*	33.4843
	G	**33.5203**	33.4295	-	33.4605	*28.5708*	33.4690
	B	**33.5049**	33.4856	-	31.9747	*31.2574*	33.4965

**Table 13:** Mean Squared Error (MSE) and Peak Signal to Noise Ratio (PSNR) under data loss.

Data Loss	MSE	PSNR
12.5%	1195	17.35
25%	2315	14.48
50%	4502	11.60

**Table 14:** MSE and PSNR under salt and pepper noise.

Salt and Pepper Noise	MSE	PSNR
1%	610	20.28
5%	2684	13.84
10%	4490	11.61

**Table 15:** Running time (unit: second).

Image Size	Encryption	Decryption
64×64×3	0.59	0.44
128×128×3	2.39	1.77
256×256×3	9.36	6.96
512×512×3	37.54	28.49

## Data Availability

Data available on request.

## Abbreviations

The following abbreviations are used in this manuscript:

6D	6 Dimensional
RNA	Ribonucleic acid
HCZRNA	Hyper-chaotic color image encryption mechanism based
	on transformed Zigzag diffusion and RNA operations
RGB	Red Greeen Blue

## References

[1] Sneha P.S., Sankar S., Kumar A.S. (2020). A chaotic colour image encryption scheme combining Walsh–Hadamard transform and Arnold–Tent maps. J. Ambient. Intell. Humaniz. Comput..

[2] Wang H., Xiao D., Chen X., Huang H. (2018). Cryptanalysis and enhancements of image encryption using combination of the 1D chaotic map. Signal Process..

[3] Jeng F.G., Huang W.L., Chen T.H. (2015). Cryptanalysis and improvement of two hyper-chaos-based image encryption schemes. Signal Process. Image Commun..

[4] Jithin K.C., Sankar S. (2020). Colour image encryption algorithm combining Arnold map, DNA sequence operation, and a Mandelbrot set. J. Inf. Secur. Appl..

[5] Zhang Y. (2018). Test and verification of AES used for image encryption. 3D Res..

[6] Xian Z.H., Sun S.L. (2010). Image Encryption Algorithm Based on Chaos and S-Boxes Scrambling. Adv. Mater. Res..

[7] Zhou Y., Hua Z., Pun C.M., Chen C.L.P. (2015). Cascade Chaotic System With Applications. IEEE Trans. Cybern..

[8] Liu L., Wang Y.N., Hou L., Feng X.R. (2017). Easy encoding and low bit–error–rate chaos communication system based on reverse–time chaotic oscillator. IET Signal Process..

[9] Zhang L., Liao X., Wang X. (2005). An image encryption approach based on chaotic maps. Chaos Solitons Fractals.

[10] Bouslehi H., Seddik H. (2018). Innovative image encryption scheme based on a new rapid hyperchaotic system and random iterative permutation. Multimed. Tools Appl..

[11] Zhang Y., Wen W., Su M., Li M. (2014). Cryptanalyzing a novel image fusion encryption algorithm based on DNA sequence operation and hyper-chaotic system. Optik.

[12] Mohammad Seyedzadeh S., Mirzakuchaki S. (2012). A fast color image encryption algorithm based on coupled two-dimensional piecewise chaotic map. Signal Process..

[13] Li T., Shi J., Zhang D. (2021). Color image encryption based on joint permutation and diffusion. J. Electron. Imaging.

[14] Askar S.S., Karawia A.A., Alshamrani A. (2015). Image Encryption Algorithm Based on Chaotic Economic Model. Math. Probl. Eng..

[15] Shaikh N., Chapaneri S., Jayaswal D. Hyper chaotic color image cryptosystem. Proceedings of the 2016 IEEE International Conference on Advances in Computer Applications (ICACA).

[16] Li C., Zhao F., Liu C., Lei L., Zhang J. (2019). A Hyperchaotic Color Image Encryption Algorithm and Security Analysis. Secur. Commun. Netw..

[17] Zhou Y., Bao L., Chen C.P. (2014). A new 1D chaotic system for image encryption. Signal Process..

[18] Kadir A., Aili M., Sattar M. (2017). Color image encryption scheme using coupled hyper chaotic system with multiple impulse injections. Opt. Int. J. Light Electron Opt..

[19] Li C., Zhang L.Y., Ou R., Wong K.W., Shu S. (2012). Breaking a novel colour image encryption algorithm based on chaos. Nonlinear Dyn..

[20] Xingyuan W., Junjian Z., Guanghui C. (2019). An image encryption algorithm based on ZigZag transform and LL compound chaotic system. Opt. Laser Technol..

[21] Xu X., Feng J. Research and Implementation of Image Encryption Algorithm Based on Zigzag Transformation and Inner Product Polarization Vector. Proceedings of the 2010 IEEE International Conference on Granular Computing.

[22] Li Y., Li X., Jin X., Zhao G., Ge S., Tian Y., Zhang X., Zhang K., Wang Z., Niu W., Li G., Liu J., Tan J., Guo L., Han Z., Batten L. (2015). An Image Encryption Algorithm Based on Zigzag Transformation and 3-Dimension Chaotic Logistic Map. Applications and Techniques in Information Security.

[23] Wang X., Guan N. (2020). A novel chaotic image encryption algorithm based on extended Zigzag confusion and RNA operation. Opt. Laser Technol..

[24] Feixiang Z., Mingzhe L., Kun W., Hong Z. (2021). Color image encryption via Hénon-zigzag map and chaotic restricted Boltzmann machine over Blockchain. Opt. Laser Technol..

[25] Sahasrabuddhe A., Laiphrakpam D.S. (2021). Multiple images encryption based on 3D scrambling and hyper-chaotic system. Inf. Sci..

[26] Hu T., Liu Y., Gong L.H., Ouyang C.J. (2017). An image encryption scheme combining chaos with cycle operation for DNA sequences. Nonlinear Dyn..

[27] Li T., Yang M., Wu J., Jing X. (2017). A novel image encryption algorithm based on a fractional-order hyperchaotic system and DNA computing. Complexity.

[28] Liu Y., Wang J., Fan J., Gong L. (2016). Image encryption algorithm based on chaotic system and dynamic S-boxes composed of DNA sequences. Multimed. Tools Appl..

[29] Chai X., Fu X., Gan Z., Lu Y., Chen Y. (2019). A color image cryptosystem based on dynamic DNA encryption and chaos. Signal Process..

[30] Hu T., Liu Y., Gong L.H., Guo S.F., Yuan H.M. (2017). Chaotic image cryptosystem using DNA deletion and DNA insertion. Signal Process..

[31] Wu J., Shi J., Li T. (2020). A novel image encryption approach based on a hyperchaotic system, pixel-level filtering with variable kernels, and DNA-level diffusion. Entropy.

[32] Liu P., Zhang T., Li X. (2019). A new color image encryption algorithm based on DNA and spatial chaotic map. Multimed. Tools Appl..

[33] Mahmud M., ur Rahman A., Lee M., Choi J.Y. (2020). Evolutionary-based image encryption using RNA codons truth table. Opt. Laser Technol..

[34] Abbasi A.A., Mazinani M., Hosseini R. (2020). Chaotic evolutionary-based image encryption using RNA codons and amino acid truth table. Opt. Laser Technol..

[35] Yadollahi M., Enayatifar R., Nematzadeh H., Lee M., Choi J.Y. (2020). A novel image security technique based on nucleic acid concepts. J. Inf. Secur. Appl..

[36] Li T., Shi J., Li X., Wu J., Pan F. (2019). Image encryption based on pixel-level diffusion with dynamic filtering and DNA-level permutation with 3D Latin cubes. Entropy.

[37] Mezatio B.A., Motchongom Tingue M., Kengne R., Tchagna Kouanou A., Fozin Fonzin T., Tchitnga R. (2020). Complex dynamics from a novel memristive 6D hyperchaotic autonomous system. Int. J. Dyn. Control..

[38] Li T., Zhou M. (2016). ECG Classification Using Wavelet Packet Entropy and Random Forests. Entropy.

[39] Li T., Qian Z., He T. (2020). Short-term load forecasting with improved CEEMDAN and GWO-based multiple kernel ELM. Complexity.

[40] Deng W., Shang S., Cai X., Zhao H., Song Y., Xu J. (2021). An improved differential evolution algorithm and its application in optimization problem. Soft Comput..

[41] Song Y., Wu D., Deng W., Gao X., Li T., Zhang B., Li Y. (2021). MPPCEDE: Multi-population parallel co-evolutionary differential evolution for parameter optimization. Energy Conv. Manag..

[42] Li T., Zhou M., Guo C., Luo M., Wu J., Pan F., Tao Q., He T. (2016). Forecasting crude oil price using EEMD and RVM with adaptive PSO-based kernels. Energies.

[43] Deng W., Xu J., Cai X., Song Y., Zhao H. (2021). Differential evolution algorithm with wavelet basis function and optimal mutation strategy for complex optimization problem. Appl. Soft. Comput..

[44] Liu L., Zhang L., Jiang D., Guan Y., Zhang Z. (2019). A Simultaneous Scrambling and Diffusion Color Image Encryption Algorithm Based on Hopfield Chaotic Neural Network. IEEE Access.

[45] Pak C., Huang L. (2017). A new color image encryption using combination of the 1D chaotic map. Signal Process..

[46] Mohamed H.G., ElKamchouchi D.H., Moussa K.H. (2020). A Novel Color Image Encryption Algorithm Based on Hyperchaotic Maps and Mitochondrial DNA Sequences. Entropy.

[47] Alvarez G., Li S. (2006). Some basic cryptographic requirements for chaos-based cryptosystems. Int. J. Bifurc. Chaos.

[48] Özkaynak F. (2018). Brief review on application of nonlinear dynamics in image encryption. Nonlinear Dyn..

[49] Murillo-Escobar M.A., Meranza-Castillón M.O., López-Gutiérrez R.M., Cruz-Hernández C. (2019). Suggested Integral Analysis for Chaos-Based Image Cryptosystems. Entropy.

